# The salmonid myostatin gene family: a novel model for investigating mechanisms that influence duplicate gene fate

**DOI:** 10.1186/1471-2148-12-202

**Published:** 2012-10-08

**Authors:** Casey B Lawson, Takumu Niino, Russell A Hermansen, Vera Brok-Volchanskaya, Melissa F Jackson, Dilip K Garikipati, David A Liberles, Buel D Rodgers

**Affiliations:** 1 Department of Animal Sciences, Washington State University, Pullman, WA 99164, USA; 2 School of Molecular Biosciences, Washington State University, Pullman, WA, 99164, USA; 3 Washington Center for Muscle Biology, Washington State University, Pullman, WA, 9916, USA; 4 Department of Molecular Biology, University of Wyoming, Laramie, WY 82071, USA

**Keywords:** Salmon, Myostatin, Gene duplication, Subfunctionalization

## Abstract

**Background:**

Most fishes possess two paralogs for myostatin, a muscle growth inhibitor, while salmonids are presumed to have four: *mstn1a, mstn1b, mstn2a* and *mstn2b*, a pseudogene. The mechanisms responsible for preserving these duplicates as well as the depth of *mstn2b* nonfunctionalization within the family remain unknown. We therefore characterized several genomic clones in order to better define species and gene phylogenies.

**Results:**

Gene organization and sequence conservation was particularly evident among paralog groupings and within salmonid subfamilies. All *mstn2b* sequences included in-frame stop codons, confirming its nonfunctionalization across taxa, although the indels and polymorphisms responsible often differed. For example, the specific indels within the *Onchorhynchus tshawytscha* and *O. nerka* genes were remarkably similar and differed equally from other *mstn2b* orthologs. A phylogenetic analysis weakly established a *mstn2b* clade including only these species, which coupled with a shared 51 base pair deletion might suggest a history involving hybridization or a shared phylogenetic history. Furthermore, *mstn2* introns all lacked conserved splice site motifs, suggesting that the tissue-specific processing of *mstn2a* transcripts, but not those of *mstn2b*, is due to alternative *cis* regulation and is likely a common feature in salmonids. It also suggests that limited transcript processing may have contributed to *mstn2b* nonfunctionalization.

**Conclusions:**

Previous studies revealed divergence within gene promoters while the current studies provide evidence for relaxed or positive selection in some coding sequence lineages. These results together suggest that the salmonid myostatin gene family is a novel resource for investigating mechanisms that regulate duplicate gene fate as paralog specific differences in gene expression, transcript processing and protein structure are all suggestive of active divergence.

## Background

The manipulation of striated muscle size and growth has several potential applications in agriculture and medicine [1]. Such advances could help treat patients with muscular dystrophy, cancer cachexia, age-related sarcopenia and/or heart failure and in addition, improve livestock production [2-4]. Many developing technologies, those that either actively enhance striated muscle growth or those that screen for polymorphisms associated with enhanced growth, target myostatin; a potential endocrine as well as local inhibitor [5,6]. Indeed, attenuating myostatin experimentally creates a “double muscling” phenotype that also occurs in *mstn−/−* animals and in those overexpressing dominant-negative receptors or one of several known myostatin binding proteins [1].

The fundamental mechanisms of myostatin action in mammals are well known, but have only recently been described in other vertebrates, particularly fish [1]. The myokine appears to inhibit muscle progenitor cell proliferation in all systems, although studies with mammalian cell lines and primary fish myosatellite cells suggest it either inhibits or stimulates differentiation, respectively [7-11]. This discrepancy is partially explained by culture conditions and by the immortalized phenotype of cell lines. Nevertheless, it is one of several ways that myostatin biology differs between mammals and fish.

In fact, most fish species possess two distinct myostatin genes [12,13] that were retained after an early genome duplication, specifically in ray-finned (Actinopterygii) fishes, over 300 Ma ago [14,15]. The more recent tetraploidization of modern salmonids, approximately 25–100 Ma ago, produced four myostatin paralogs (*mstn1a, mstn1b, mstn2a & mstn2b*), although *mstn2b* is a pseudogene in rainbow trout [16]. Each paralog is differentially expressed in rainbow trout and the *mstn2* transcripts are alternatively processed in a way that contributes to the nonfunctionalization of *mstn2b* and to the tissue-specific actions of *mstn2a*. The differential patterns of gene expression and transcript processing among the rainbow trout MSTN paralogs suggests that subfunctionalization (complementary & compartmentalized function), neofunctionalization (adopting new functions) and nonfunctionalization may have all contributed to this gene family’s evolution. A better understanding of myostatin gene structure and phylogenies among other salmonids would therefore help explain fundamental mechanisms that influence duplicate gene fate and ultimately control their fixation and maintenance, which in turn enables the functional diversification of genes and genomes [17].

The evolution of myostatin has previously been studied among mammalian orthologs, where it was found to be broadly conserved, except for periods of rapid sequence evolution in ruminant Artiodactyls [18]. Because of the suggested link between gene duplication and functional divergence, myostatin was studied within the salmonids. Reported herein is the structural characterization and phylogenetic analysis of 33 MSTN paralogs cloned from several species within the three salmonid subfamilies: Salmoninae, Coregonidae and Thymallidae, with Retropinna as an outgroup species. The results together describe a unique and novel gene family model for examining not only salmonid phylogenies, but also different molecular mechanisms that influence duplicate gene fate.

## Results

### Comparative Mapping and Gene Organization

**Figure 1:**
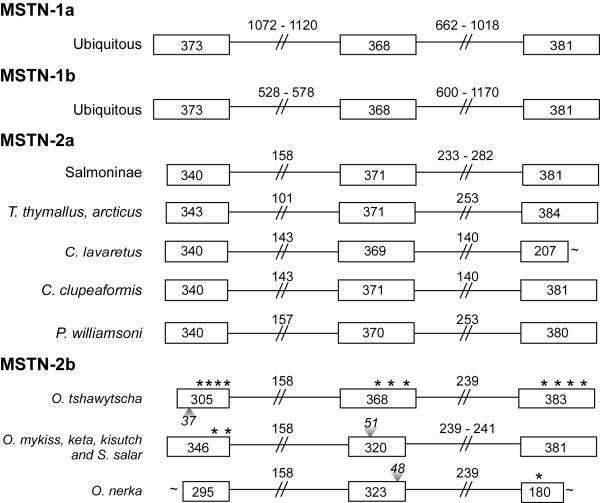
**Comparative mapping of coding and non-coding sequences of salmonid myostatin paralogs.** The genomic structure and organization (5’ to 3’) of MSTN-1a, -1b, -2a, and -2b are divided into three exons (boxed) connected by two introns (intervening lines) with the number of basepairs (bp) indicated for each (~, unsequenced 5’ or 3’ regions). Species/taxa are indicated on the left under headings for each paralog grouping (ubiquitous, common to all orthologs). Regions missing within a particular paralog group (number of bp shown), but present in the others, are labeled with gray arrowheads and in-frame stop codons are labeled with asterisks.

The basic organization of each salmonid myostatin paralog is highly conserved as each gene contains three exons (Figure1). This is true not only in salmonids, but in all other vertebrates as well [1]. Gene structures are most similar among the *mstn1* genes, as indicated by conserved exon lengths, although the *mstn2a* genes differed in length by only 1–3 bp. Most variability understandably occurred within introns and these differences were reflected in the taxa, particularly among the *mstn2a* genes. Intron sizes were also hierarchal as in general, *mstn1a* introns were largest followed by those of *mstn1b* and the *mstn2* genes. Thus, differences in intron and exon size alone can often be used to distinguish individual paralogs, even if not computing molecular phylogenies. 

*Mstn2b* paralogs were only cloned from species within the Salmoninae subfamily and in every case, each was a pseudogene. Nonfunctionalization appears to have arisen independently among these genes as the in-frame stop codons occurred in different locals. This could also be due to mutations that occurred after nonfunctionalization, although a closer examination of the specific indels suggests this is not the case (see below). Three notable deletions include 37 bp from the *O. tshawytscha* first exon, 48 bp from the *O. nerka* second exon and 51 bp from the *O. mykiss*, *O. keta*, *O. kisutch* and *S. salar* second exons. The fact that the latter 51 bp region is missing in *S. salar* and is retained in *O. tshawytscha* and *O. nerka* suggests that a common ancestor to these latter two species diverged well before the more recent salmonid radiation. This is supported by the similar distribution of stop codons, and underlying molecular changes (see below), as well as the retention of the aforementioned 17 and 48 bp regions in several other *Oncorhynchus* species and in *S. salar*. Pseudogenes evolve randomly and are not influenced by selection pressures at the protein or expression levels. Therefore, the signal in these pseudogenes will not convolute ancestral and functional (sometimes convergent) signal as is prone to happen in gene family evolution.

### Multiple sequence alignments

**Figure 2:**
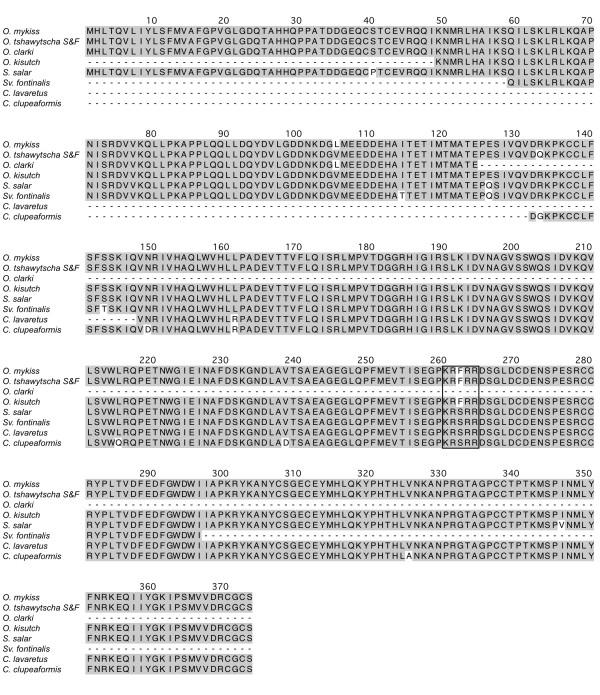
**Multiple sequence alignment of salmonid MSTN-1a paralogs.** Amino acid positions are numbered above the sequence line, taxa are indicated to the left and amino acid identities are shaded gray. Gaps and unsequenced regions are indicated by dashes and the furin/PC cleavage site is boxed.

**Figure 3:**
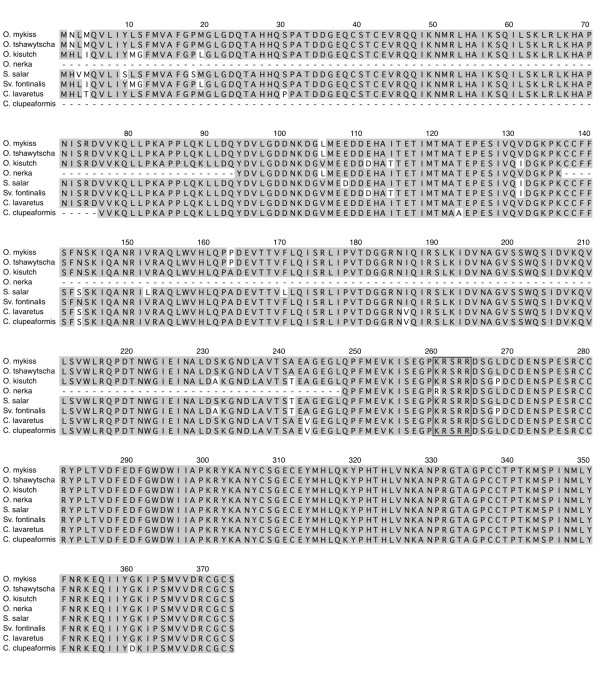
**Multiple sequence alignment of salmonid MSTN-1b paralogs.** Amino acid positions are numbered above the sequence line, taxa are indicated to the left and amino acid identities are shaded gray. Gaps and unsequenced regions are indicated by dashes and the furin/PC cleavage site is boxed.

**Figure 4:**
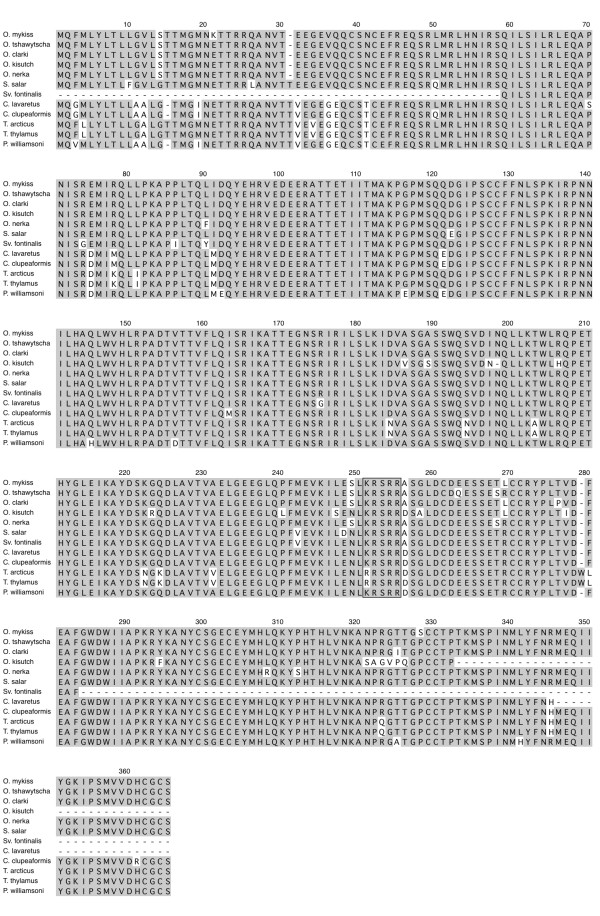
**Multiple sequence alignment of salmonid MSTN-2a paralogs**. Amino acid positions are numbered above the sequence line, taxa are indicated to the left and amino acid identities are shaded gray. Gaps and unsequenced regions are indicated by dashes and the furin/PC cleavage site is boxed.

The coding region for each orthologous group is well conserved as individual comparison between any two salmonid species indicated that the MSTN-1a, -1b and -2a proteins are on average 99%, 98% and 92% identical, respectively (Figures2, 3, and 4). Several taxa-specific features were identified and could aid in determining gene family phylogenies. These include F262 that occurs in all *Oncorhynchus* MSTN-1a proteins (Figure3). This substitution is particularly noteworthy as it lies within the furin/prohormone convertase (PC) recognition sequence that is necessary for the cleavage and formation of mature myostatin peptide [1]. In addition, V243 is common only to the Coregonid MSTN-1b sequences (Figure3) and 27 unique positions were identified among the different MSTN-2a sequences (Figure4). This includes a 7 bp deletion in exon 1 of *Sv. fontinalis mstn2a* that produces a frame shift, although an alternative upstream initiator results in a complete open reading frame that is very similar to the other MSTN-2a amino acid sequences (Figure4). This pattern of conservation and divergence is reminiscent to that of intron sizes as it reflects a hierarchy where the coding sequences are most conserved among MSTN-1 paralogs and most divergent among the MSTN-2a. 

**Figure 5:**
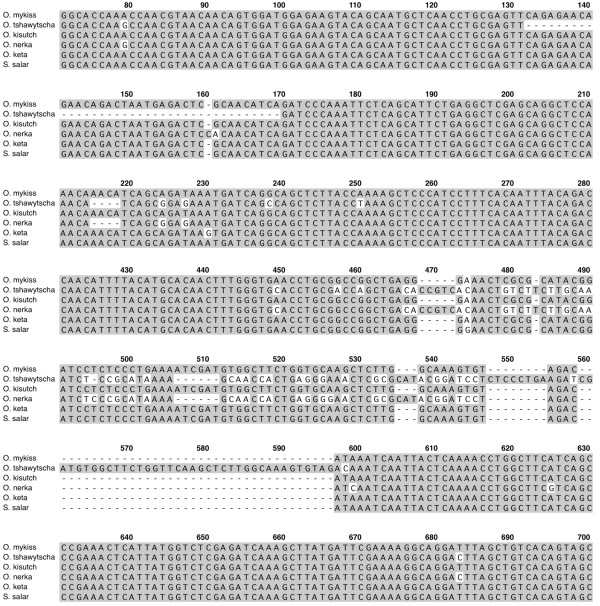
**Multiple sequence alignment of salmonid MSTN-2b cDNA.** Nucleotide positions are numbered above the sequence line, taxa are indicated to the left and identities are shaded gray. Gaps and unsequenced regions are indicated by dashes. Note that only selected regions are shown as the number line is discontinuous.

Comparing the MSTN-2b cDNA sequences indicated, as with the gene organization data, that the *O. tshawytscha* and *O. nerka* genes are most similar. Each lack a 4 bp region at position 215–218 that is common to the other *Oncorhynchus* species and to *S. salar* (Figure5). They also share a 5 bp insertion at position 468–472 and an adjacent downstream region of 73 bp that differs significantly from the other orthologs in addition to several other single nucleotide polymorphisms. Furthermore, the *O. tshawytscha* sequence lacks a 37 bp cassette at position 132–169 and contains an additional 46 bp between 557–596. The former shifts the coding frame and is responsible for producing many stop codons (Figure1). In all other sequences, excluding *O. nerka*, a single bp is missing at position 160, which introduces a frame shift and several premature stop codons. With an intact 1st exon coding region (e.g. no 37 bp deletion), the *O. nerka* coding frame changes multiple times before introducing a stop codon in the 3rd exon that is located after the region coding for the furin/PC site (data not shown). This opens the possibility that a truncated and mutated myostatin prodomain (a.k.a. latency associated protein, LAP) for MSTN-2b could be produced in *O. nerka*. An assessment of motifs necessary for mRNA processing, however, suggests that this does not occur (see below). The similarities noted between *O. tshawytscha* and *O. nerka* sequences are together suggestive of a common ancestor that diverged early from other salmonids. The notable differences, however, likely occurred after the two species subsequently diverged from the ancestor. Nevertheless, these data could prove useful in reevaluating salmonid phylogenetic relationships.

### In silico *assessment of mRNA splice site motifs*

**Figure 6:**
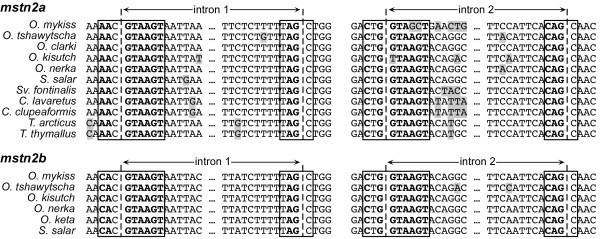
**Exon/intron boundaries for MSTN-2a and -2b genes.** The 5’ and 3’ sequences of introns 1 and 2 are shown with exon/intron boundaries indicated by dashed lines. Splice site sequences are boxed and nucleotides consistent with the known consensus requirements for 5’ (RAG/GTRAGT; R = A or G) and 3’ (CAG/G) splice sites are in bold. Polymorphisms within each orthologous group are shaded.

Rainbow trout *mstn2* transcripts are alternatively spliced in a manner that prevents the production of mature *mstn2b* transcripts in all tissues and limits *mstn2a* processing to specific tissues and developing conditions [10,11,16]. We therefore assessed exon/intron boundaries and identified putative branch points in each *mstn2* gene to determine the likelihood of transcript processing across taxa. None of the boundaries, regardless of gene, possessed an intact splice site (Figure6). At least one of the nucleotides flanking each boundary, those most critical for splicing, were mutated in all genes except for the 5’ boundary of the second introns. Putative branch point motifs were identified in the first introns of all genes, although by contrast, these motifs were either missing or mutated in the second introns (Table1). These data strongly suggest that the alternative processing of *mstn2* transcripts is a common feature among Salmoninae species if not all salmonids. The boundary sequences were remarkably similar in all genes indicating that the underlying changes occurred in a basal salmonid species, but after tetraploidization as all *mstn1* transcript processing sites are functionally intact. Thus, alterations in transcript processing may have precluded changes in coding sequences that together contributed to the divergence of *mstn2a* and nonfunctionalization of *mstn2b*. 

**Table 1:** **Putative branch points in salmonid *mstn2 *introns**

**species**	***mstn2a *Intron 1**	***mstn2a *Intron 2**	***mstn2b *Intron 1**	***mstn2b *Intron 2**
**O. mykiss**	**NOT FOUND**	**G**GCTGAC 919 (50)	**TTCTAAC 484 (138)**	**NOT FOUND**
		TACTAAT 955 (86)		
O. tshawytscha	**A**ACTGAC 457 (117)	**G**GCTGAC 982 (23)	TTCTAAC 443 (138)	NOT FOUND
	TTCTAAC 478 (138)			
*O. clarki*	TTCTAAC 478 (138)	**G**GCTGAC 891 (23)		
*O. kisutch*	TTCTAAC 479 (139)	NOT FOUND	TTCTAAC 484 (138)	NOT FOUND
*O. keta*			TTCTAAC 484 (138)	NOT FOUND
*O. nerka*	TTCTAAC 478 (138)	**G**GCTGAC 892 (23)	TTCTAAC 433 (138)	NOT FOUND
*S. salar*	TTCTAAC 481 (138)	**G**GCTGAC 895 (23)	TTCTAAC 484 (138)	NOT FOUND
*Sv. fontinalis*	TTCTAAC 361 (138)	NOT FOUND		
*C. lavaretus*	TCCTAAC 463 (123)	NOT FOUND		
*C. clupeaformis*	TTCTAAC 463 (123)	NOT FOUND		
*T. arcticus*	TTCTAAC 424 (81)	NOT FOUND		
*T. thymallus*	TTCTAAC 424 (81)	NOT FOUND		

Polymorphysms in the known consensus (TNCTRAY; N = any nucleotide, R = A or G, Y = C or T) are bold. Numbers represent the distance (bp) of the first nucleotide within putative sites from the initiator or the 5’ intron boundary (parenthesis). In some instances, two putative branch points were identified.

### Phylogenetic analysis

**Figure 7:**
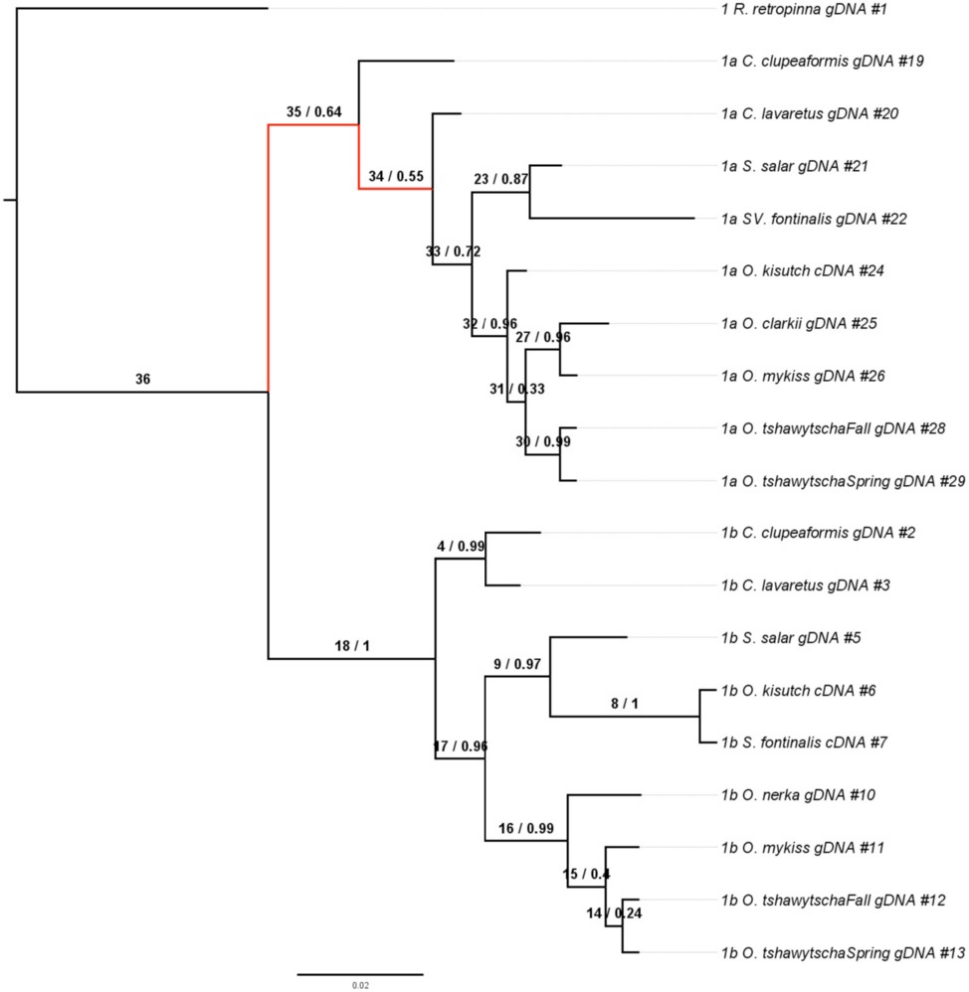
**Phylogeny of MSNT-1a and -1b genes.** The topology for the myostatin 1a and 1b genes is shown here. The phylogeny was constructed using MrBayes 3.2 [47]. The tree was rooted with *R. retropinna.* Branches colored in red showed a statistically significant dN/dS value for the foreground branch over the background as indicated in Table 4. Branches are labeled as the foreground branch followed by the posterior probability of the branch.

**Figure 8:**
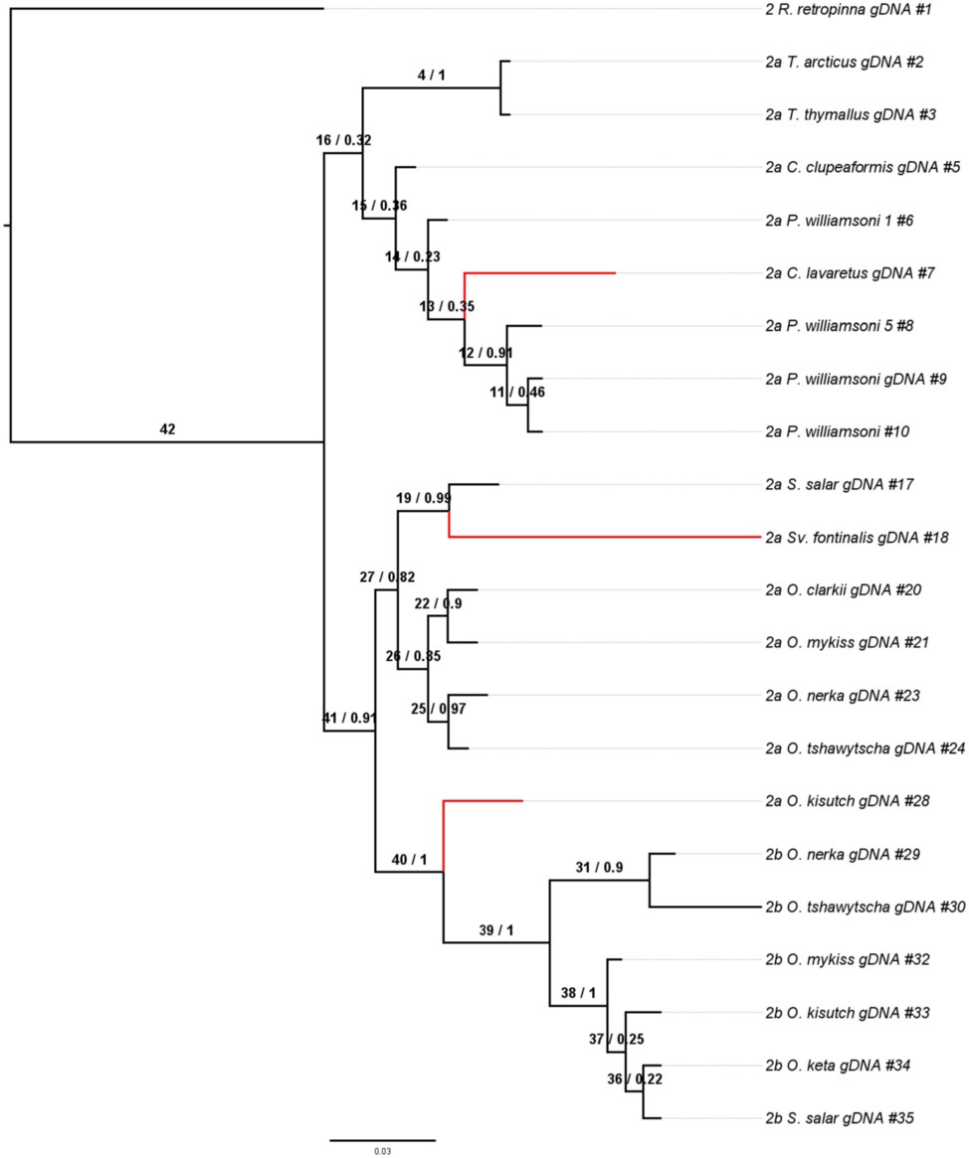
**Phylogeny of MSNT-2a and -2b genes.** The topology for the myostatin 2a and 2b genes is shown here. The phylogeny was constructed using MrBayes 3.2 [47]. The tree was rooted with *R. retropinna.* Branches colored in red showed a statistically significant dN/dS value for the foreground branch over the background. Branches are labeled as the foreground branch followed by the posterior probability of the branch.

Phylogenetic analysis of the myostatin genes revealed that the evolution of *mstn1a* and *mstn1b* closely resembles the established species phylogenies. The only exception was the grouping of *O. kisutch mstn1b* with *S. fontinalis* instead of other *Oncorhynchus* species (Figure7)*.* This exception was strengthened in the *mstn1a* tree estimated through MrBayes, which showed *O. kisutch* as the first genus to diverge and therefore might be the oldest member of the clade. The *Oncorhynchus* clade in the amino acid tree was poorly resolved by Phyml and little inference could be gained, however *O. kisutch* was placed as an outgroup to the clade by the ML method when using the DNA sequences with high bootstrap support. This relationship was strongly conserved in all of the trees for *mstn1b*, produced through either a ML or Bayesian estimate. The *mstn2* (Figure8) phylogenies revealed more about the possible relationships between salmonids and in general, they appear to have diverged in accordance with the believed species tree, although with two notable exceptions. Firstly, *O. kisutch* seems to have experienced more change than the rest of *Oncorhynchus* as demonstrated by the placement of *O. kisutch mstn2a* outside of the other *Oncorhynchus mstn2a* orthologs (Figure8). This again suggests that it diverged earlier and has had more time to evolve. Secondly, there was weak support for an *O. tshawytscha* and *O. nerka* clade in the *mstn2b* tree at the DNA level. The *mstn1b* and *mstn2a* trees, by contrast, suggest evidence of an older common ancestor that separates the two taxa. Nevertheless, there was stronger support at the amino acid level as trees for MSTN-1b, MSTN-2a and MSTN-2b (Additional files 1 &2) indicate the relationship between *O. tshawytscha* and *O. nerka* is upheld with bootstrap support. Further, signal in the substitution data is complemented by the observation of shared rare indel events that are unlikely to have occurred independently by chance. A complete tree showing all MSTN1 and MSTN2 genes was generated using ML and Bayesian methods and can be seen in the supplementary data (Additional file 3).

**Figure 9:**
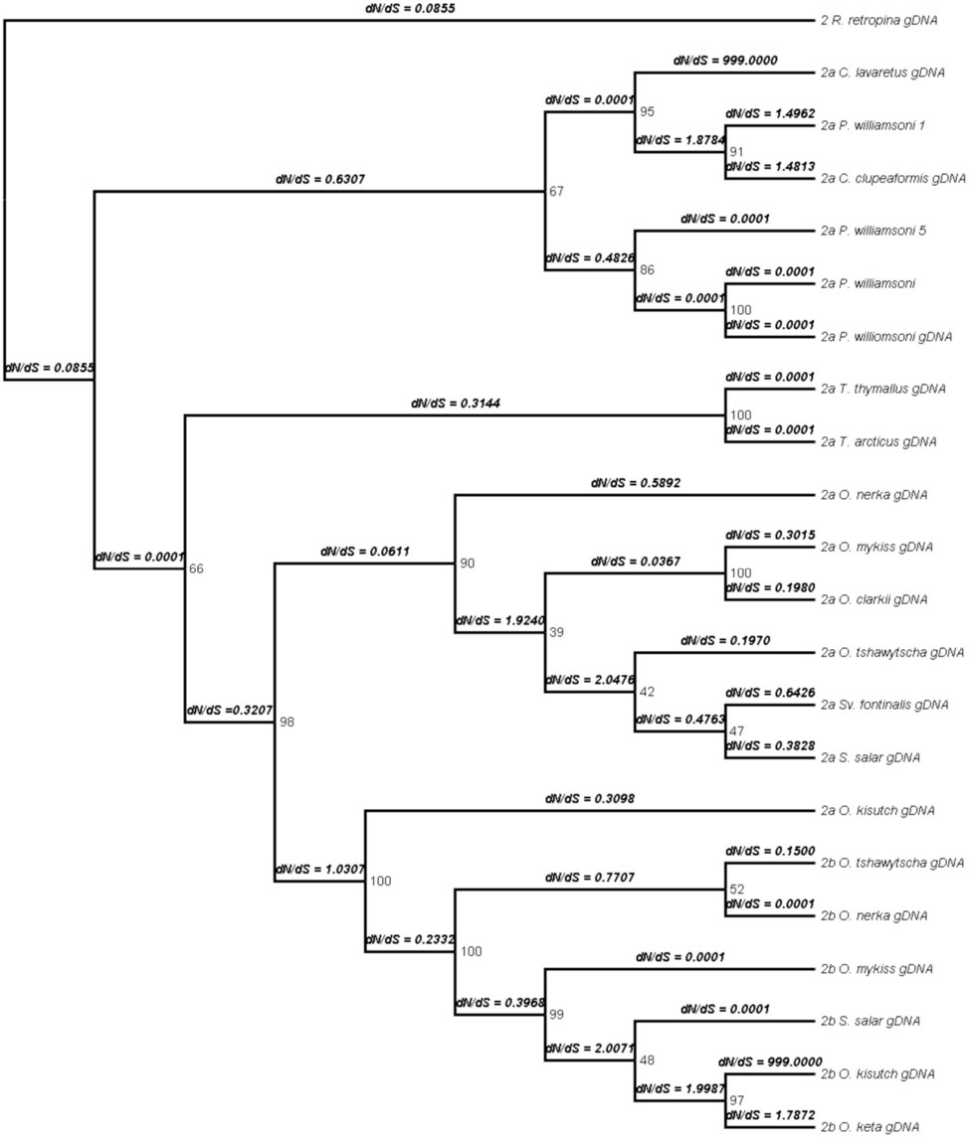
**Analysis for positive selection.** Cladogram of the MSTN-2a, -2b tree. dN/dS ratios were calculated along each branch using the free ratios model. The tree showed several possible locations of positive selection and increased rate variation where the dN/dS ratios were much greater than 1. The dN/dS analysis was not performed on the MSTN-1a, -1b tree because of lack of support for a free-ratios model.

**Figure 10:**
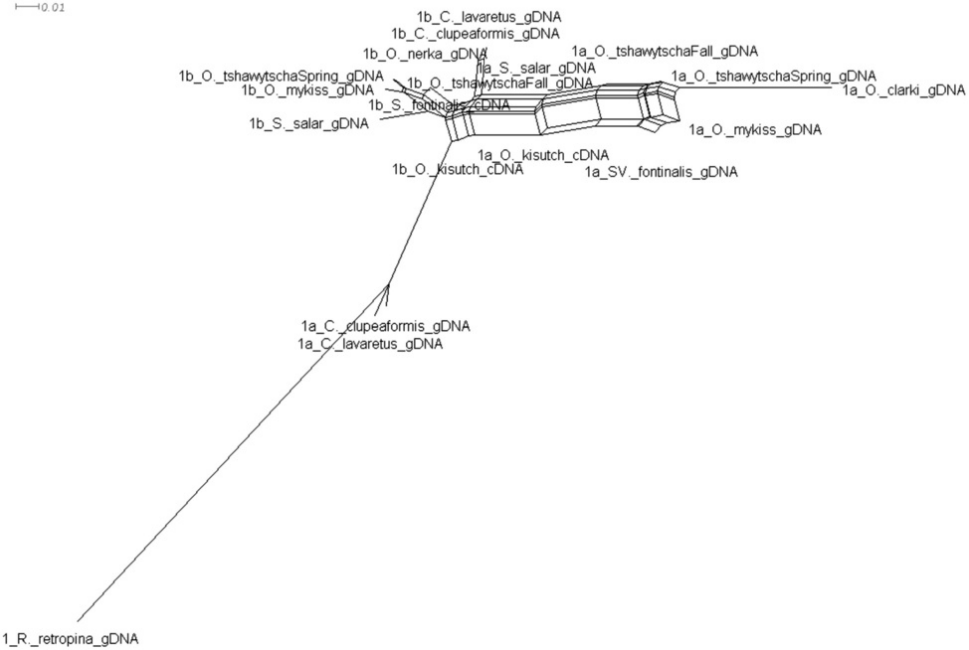
**Splitstree for MSTN-1a, -1b.** Splitstree built using SplitsTree4. The tree represents the possible amount of hybridization that has occurred within MSTN-1a, -1b in salmonids.

**Figure 11:**
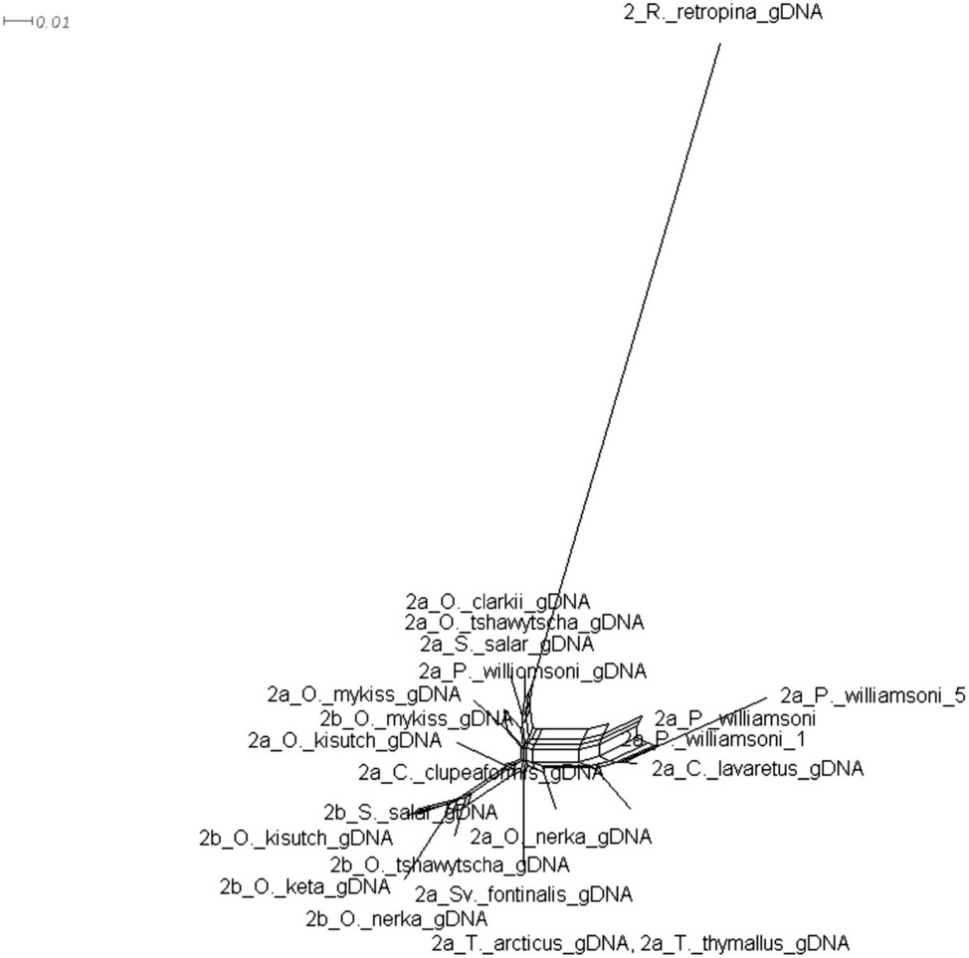
**Splitstree for MSTN-2a, -2b.** Splitstree built using SplitsTree4. The tree represents the possible amount of hybridization that has occurred within MSTN-2a, -2b in salmonids.

Evidence for variable rate changes (and corresponding selection) along different branches was detected in the MSTN-1a/1b and MSTN-2a/2b trees by measuring dN/dS ratios using both a branch and a branch-site analysis. In the branch-site analysis (Tables 2, 3), all branches displaying a statistically significant dN/dS value greater than 1 are locations with substantial increases in rate variation and are possible sites for relaxed selective constraint or positive selection. Within the MSTN-1a/1b tree, two branches showed evidence of variable rate change, most notably separating the *mstn1a* genes from the *mstn1b* genes. The MSTN-2a/2b tree also showed signals of relaxed selective constraint, with four branches demonstrating a statistically significant dN/dS value greater than 1. Interestingly, one of the four branches showing a signal for dN/dS was the branch leading to *O. kisutch mstn2a*, which was placed outside of all of the other *mstn2a* genes, further strengthening the conclusion that this gene has evolved differently than other *mstn2a* genes. The Bayes Empirical Bayes (BEB) analysis revealed one site within the MSTN-1a/1b tree under positive selection with a probability greater than 95 (Additional file 4). This site, position 185 on branch 34 is located within the propeptide (positions 24–266) of the protein. The MSTN-2a/2b analysis revealed several sites under positive selection all located within the propeptide (Additional file 4) located on branches 7, 18, and 19. None of the sites (listed in Additional file 4) determined to be under positive selection correlated with the sites found by Tellgren, *et al.*, 2004 for orthologous divergence in ruminant Artiodactyls.Within the branch model of MSTN-2a/2b (Figure9, dN/dS values calculated across each branch in Figure8), eleven branches had values greater than 1, although the test does not allow for establishing individual branches as being significantly greater than 1. Most notable among the branches is a high signal for rate variation within the *mstn2b* phylogeny, presumably all pseudogenes, which is indicative of relaxed selection. No figure is present for the branch model of the MSTN-1a/1b tree as there was no statistical support for a free-ratios model over the single-ratio model. SplitsTree was run to test if there was strong support for a single phylogenetic tree that could explain the underlying sequence data in *mstn1* and *mstn2* (Figures10 &11). This analysis revealed highly non-tree like structures for both paralog groups and is suggestive of hybridization, incomplete lineage sorting or stochastic phylogenetic incongruence.

**Table 2:** Likelihood ratio test analysis and dN/dS ratios for MSTN-1a and MSTN-1b

**Foregroun branch**	**P-value**	**Likehood ratio test**	**p**_**0**_	**p**_**1**_	**W**_**0**_	**W**_**2**_
1	1	0	0.78604	0.0735	0.06418	1
2	1	0	0.91449	0.08551	0.06418	1
3	1	0	0.87254	0.08151	0.06349	1
4	1	0	0.84418	0.0783	0.06256	1
5	1	0	0.75124	0.07563	0.05589	1
6	1	0	0.7823	0.07315	0.06418	1.0937
7	1	0	0.78494	0.0734	0.06418	1.0519
8	0.837932705	0.041836	0.8317	0.06936	0.06127	1.53474
9	0.435190969	0.60893	0	0	0.05808	116.56426
10	1	0	0.91449	0.08551	0.06418	1
11	0.480047236	0.498756	0	0	0.05991	154.56582
12	0.998871621	2.00E-06	0.78682	0.07357	0.06418	1.05741
13	0.998871621	2.00E-06	0.78321	0.07324	0.06418	1
14	1	0	0.91449	0.08551	0.06418	1
15	1	0	0.91449	0.08551	0.06418	1
16	1	0	0.91449	0.08551	0.06418	1
17	1	0	0.91449	0.08551	0.06418	1
18	0.02608246	4.950544	0.91976	0.07657	0.06708	998.99999
19	1	0	0.77391	0.07197	0.06306	1
20	1	0	0.91449	0.08551	0.06418	1
21	0.80351773	0.0619	0	0	0.06339	104.27124
22	1	0	0.91449	0.18551	0.06418	1
23	1	0	0.7271	0.06908	0.05868	1
24	1	0	0.91149	0.08551	0.06418	1
25	1	0	0.91149	0.08551	0.06418	1
26	1	0	0.91149	0.08551	0.06418	1
27	1	0	0.91149	0.08551	0.06418	1
28	0.998871621	2.00E-06	0.78417	0.07332	0.16418	1.04859
29	0.998404232	4.00E-06	0.78127	0.07305	0.16418	1.06811
30	1	0	0.91449	0.08551	0.06418	1
31	1	0	0.77679	0.07263	0.16418	1.34502
32	1	0	0.91149	0.08551	0.16418	1
33	1	0	0.91149	0.8551	0.06418	1
***34***	***0.001154703***	***10.561454***	***0.9179***	***0.72176***	***0.06509***	***103.9286***
***35***	***0.009387076***	***10.945966***	***0.91398***	***0.07251***	***0.0627***	***999***
36	1	0	0.78794	0.07368	0.06418	1

Summary statistics generated for each foreground branch using a Branch-site test. Branches in bold and italics are are statistically significant with a dN/dS ratio greater than 1. Branch numbers are labeled in Figure 7.

**Table 3:** Likelihood ratio test analysis and dN/dS ratios for MSTN-2a and MSTN-2b

**Foreground branch**	**P-value**	**Likehood ratio test**	**p**_**0**_	**p**_**1**_	**W**_**0**_	**W**_**1**_
1	0.992953366	7.80E-05	0.85159	0.14839	0.11865	1
2	0.9953476	3.40E-05	0.85149	0.14837	0.11865	1
3	0.9953476	3.40E-05	0.85148	0.14837	0.11865	1
4	1	0	0.60715	0.10719	0.10494	1
5	1	0	0.70568	0.12296	0.11865	1.04992
6	0.998871621	2.00E-06	0.7009	0.12213	0.11865	1.04909
7	3.42E-11	43.922434	0.8387	0.12582	0.11621	518.94859
8	1	0	0.85161	0.14839	0.11865	1
9	1	0	0.85161	0.14839	0.11865	1
10	0.993717516	6.20E-05	0.8516	0.14839	0.11865	1
11	1	0	0.85161	0.14839	0.11865	1
12	1	0	0.69073	0.12412	0.11458	1
13	1	0	0.6942	0.12096	0.11862	1.20916
14	1	0	0.70098	0.1214	0.11862	1.05347
15	1	0	0.85161	0.14839	0.11865	1
16	0.625942013	0.237604	0.70371	0.12262	0.11865	1.12316
17	1	0	0.30086	0.05619	0.10976	1
***18***	***1.70E-12***	***49.80162***	***0.83059***	***0.09738***	***0.11719***	***146.51077***
***19***	***0.010178788***	***6.603336***	***0.83919***	***0.14111***	***0.11848***	***999***
20	1	0	0.85161	0.14839	0.11865	1
21	1	0	0.53537	0.09578	0.11454	1
22	1	0	0.85161	0.14839	0.11865	1
23	0.6663122	0.185568	0.77072	0.13927	0.11378	5.51208
24	1	0	0.727	0.12965	0.11646	1
25	1	0	0.85161	0.14839	0.11865	1
26	1	0	0.85161	0.14839	0.11865	1
27	1	0	0.85161	0.14839	0.11865	1
***28***	***4.37E-05***	***16.704412***	***0.83602***	***0.15333***	***0.1199***	***999***
29	1	0	0.85161	0.14839	0.11865	1
30	1	0	0.85161	0.14839	0.11865	1
31	1	0	0.85161	0.14839	0.11865	1
32	1	0	0.70563	0.12296	0.11865	1.08115
33	0.460406603	0.544906	0	0	0.11556	140.21885
34	0.994358151	5.00E-05	0.70444	0.12275	0.11865	1.06825
35	1	0	0.85161	0.14839	0.11865	1
36	1	0	0.70412	0.12269	0.11865	1.05
37	1	0	0.70542	0.12292	0.11865	1.05032
38	1	0	0.85161	0.14839	0.11865	1
39	1	0	0.69477	0.12223	0.11202	1
40	1	0	0	0	0.10345	1
41	1	0	0.74051	0.12707	0.11695	1
42	1	0	0.73126	0.12428	0.11865	1

Summary statistics generated for each foreground branch using a Branch-site test. Branches in bold and italics are statistically significant with a dN/dS ratio greater than 1. Compare foreground branches with Figures 8, 9.

## Discussion

Gene duplication is an important process that alters gene function often via changes in gene structure/sequence, patterns of gene expression and as recently determined, altered transcript processing [19-21]. Examples of such differences are seen in the salmonid myostatin gene family, suggesting that it is diverging via a combination of neofunctionalization and subfunctionalization. The former involves the introduction of new functions for duplicate genes whereas with subfunctionalization, duplicate genes specialize to form complementary functions [17]. For example, the expanded repertoire of expression patterns in the composite of fish myostatin genes relative to mammalian homologs suggests that expression profiles have neofunctionalized following the teleost genome duplication [22]. How this interplays with the second whole genome duplication in salmonids [23] is unclear.

Generally, the evolutionary dynamics of duplicate genes resulting from whole genome duplications differ from those that occur after small-scale events [24,25] and result in a higher probability of long-term retention. In fact, duplicate genes are typically retained over long evolutionary periods only after some divergence of function, which as discussed, can result from changes in gene structure, promoter activity and/or transcript processing. Gene expression profiles and subfunctionalization tend to evolve faster than coding sequence functions and neofunctionalization due to the greater potential for deleterious versus advantageous changes [26-28]. Of course, the vast majority of duplicate genes is not retained, but is rapidly nonfunctionalized, as with *mstn2b*. Most studies investigating the underlying mechanisms of nonfunctionalization have focused primarily on coding sequence mutations. Thus, the fact that changes in transcript processing contributed to the nonfunctionalization of both *mstn2* paralogs is truly novel especially as it appears to predate the coding sequence changes and results in the tissue-specific nonfunctionalization of *mstn2a*. In fact, alternative processing has only recently been demonstrated to influence gene fate [29-31], which further illustrates the importance of studying the salmonid myostatin gene family. Future studies are nevertheless needed to confirm that the *mstn2a* and *mstn2b* transcript processing patterns are indeed conserved among all salmonids. Nevertheless, the shared lack of intact motifs necessary for removing intronic sequences, in *mstn2* paralogs cloned from all three subfamilies, suggests that the patterns are indeed conserved among all salmonids.

We recently demonstrated subfunctionalization of rainbow trout *mstn1a* and *mstn2a* specifically in their ability to regulate the differentiation of primary myosatellite cells [11]. In mammals, myostatin is upregulated in differentiating muscle cells and by insulin-like growth factor (IGF)-I [32-35], a known endocrine regulator of myogenesis [36]. It is therefore believed to partly mediate the actions of IGF-I on muscle cell differentiation [1,37-39]. This represents an ancestral state where a single myostatin gene serves multiple functions. The process is more complicated in rainbow trout as *mstn1a* appears to stimulate differentiation in response to serum whereas *mstn2a* in response to IGF-I. The combined actions of a single myostatin gene in mammals have therefore subfunctionalized in rainbow trout and possibly other salmonids with regard to this function. The relative changes in *mstn1b* expression for the most part mirror those of *mstn1a*, although *mstn1a* levels always exceed those of *mstn1b*. This is suggestive of functional redundancy, at least in regards to muscle cell differentiation.

Neofunctionalization and subfunctionalization in the expression of the different paralogs is due to differences in promoter structure/function as the genes are differentially expressed by these different myogenic conditions. Alternative processing of *mstn2a* transcripts plays a role as well as it is also stimulated by IGF-I. Nova proteins are known regulators of alternative processing in the brain, the only tissue where *mstn2a* transcripts are fully processed without IGF stimulation, and recognize YCAY motifs that can either direct or misdirect the splicesome [40-42]. A survey of such motifs throughout the *mstn2a* and *mstn2b* genes identified several putative binding sites that are unique to *mstn2a*, particularly in the second intron (Table4). It is unknown whether Nova proteins truly regulate *mstn2a* processing in brain, muscle or in response to IGF-I. The suggested model of neofunctionalization or subfunctionalization via alternative processing, however, is extremely novel and the possible contributions of Nova proteins is at least plausible and best of all, testable. It is also amenable to computational approaches that could track selection events that influence transcript processing across taxa and thus, the molecular mechanisms of functional change itself. 

**Table 4:** **Number and location of YCAY motifs in salmonid *mstn2 *genes**

	***O myk***	***O ner***	***O tsh***	***O kis***	***S sal***	***O ket***	***O cla***	***Sv fon***	***T thy***	***T arc***	***C clu***	***C lav***
Ex 1	8/8	8/NA	8/7	8/8	8/8	NA/8	8	NA	9	9	8	8
In 1	4/4	4/4	4/3	4/4	4/4	NA/4	4	4	1	1	3	3
Ex 2	6/5	7/7	7/7	7/5	7/5	NA/5	6	6	6	6	6	6
In 2	13/7	13/8	12/6	7/6	13/8	NA/6	13	13	11	11	8	8
Ex 3	10/10	10/NA	10/8	7/4	9/9	NA/5	9	NA	12	11	10	NA
Total	**41/34**	**42/NA**	**41/31**	**33/27**	**41/34**	**NA/28**	**40**	**N/A**	**39**	**38**	**35**	**N/A**

Numbers are for *mstn2a*/*mstn2b*, respectively. Single numbers/cell represent *mstn2a* only. (Y, C or T; NA, not available due to incomplete sequences). Abbreviations: *O, Oncorhynchus; myk, mykiss; ner, nerka; tsh, tshawytscha; kis, kisutch; S sal, Salmo salar; ket, keta; cla, clarki; Sv fon, Salvelinus fontinalis; T, Thymallus; thy, thymallus; arc, arcticus; C, Coregonus; clu, clupeaformis; lav, lavaretus.*

Analyses of the *mstn1a*, *mstn1b,* and *mstn2a* phylogenetic relationships were generally consistent with the established salmonid relationships. The only exception was for *O. kisutch* which, based on the topologies of these coding genes, diverged before the other *Oncorhynchus* species examined (Figures7 &8, Additional files 1 &2). This was not supported by the topologies of the pseudogene *mstn2b*, which may be more meaningful. Indeed, pseudogenes are by definition non-functional and are not phenotypically expressed. Unlike most other genes, including mitochondrial, they evolve under neutral processes only and thus, phylogenetic relationships of orthologous pseudogenes are often excellent predictors of species phylogenies. The relationships defined by the *mstn1a*, *mstn1b* and *mstn2a* trees may therefore by more representative of gene functional pressures rather than species phylogenies. Several lines of evidence nevertheless support a closer relationship for *O. tshawytscha* and *O. nerka* than previously suggested. This includes a unique *mstn2b* clade (Figure8), strong support in the MSTN-1b, MSTN-2a and MSTN-2b amino acid trees (Additional files 1 &2), and shared *mstn2b* indels (Figure1). Revising salmonid phylogenetic relationships based solely on these data would clearly be premature. This is particularly true as the Splitstree data indicate a high level of hybridization or conflicting phylogenetic signal generated by other mechanisms within the family (Figures10 &11). However, these studies do suggest the need to reassess the family, possibly by including more pseudogenes as well as more representatives of the myostatin gene family.

## Conclusions

These studies together suggest that although the genomic organization of all paralogs is relatively well conserved, several notable structural differences that influence either coding sequences and/or transcript processing have indeed contributed to paralog divergence across taxa. Furthermore, the salmonid myostatin gene family appears to be actively diverging and is therefore a unique model system for investigating mechanisms that ultimately influence duplicate gene fate. Analysis of *mstn2b* structure in particular also suggests that a common ancestor to *Oncorhynchus tshawytscha* (a.k.a. king or chinook salmon) and *O. nerka* (a.k.a. sockeye salmon) diverged early and before the more recent salmonid radiation. This gene family is therefore a highly novel system for assessing gene and species phylogenies.

## Methods

### Animals & tissue handling

Genomic (g)DNA or fin clips from different salmonids was provided by the Washington State University Aquaculture Core or by collaborators (see below). This includes samples from rainbow trout (*Oncorhynchus mykiss*), cutthroat trout (*O. clarki*), sockeye salmon (*O. nerka*), Chinook salmon (*O. tshawytscha*), coho salmon (*O. kisutch*), chum salmon (*O. keta*), Atlantic salmon (*Salmo salar*), brook trout (*Salvelinus fontinalis*), greyling (*Thymallus thymallus*; from Nicola Barson, University of Oslo), arctic greyling (*T. arcticus*; from Christopher Myskiw, Fisheries & Oceans Canada), lake and mountain whitefish (*Coregonus clupeaformis* &*Prosopium williamsoni*; both from Peter Unmack, Brigham Young University), common whitefish (*C. lavaretus*) and common smelt (*Retropinna retropinna*; from Brendan Hicks, University of Waikato). Some sequences from *S. salar* and the *Sv. fontinalis* were downloaded from Genbank and accession numbers for all of the sequences used in this study, including novel sequences, are included in Table5. When fin clips were provided, gDNA was extracted by first incubating tissues in 3 ml of lysis buffer (30 mM Tris, 8 M Urea, 4% w/v Chaps, pH 8.0) containing 20 mg/ml proteinase K at 60°C. Three consecutive phenol:chloroform:isoamyl alcohol extractions were then performed and gDNA quality was verified on a 1% agarose gel. Fish were maintained in an AAALAC approved facility and samples were obtained according to animal use protocols preapproved by the universities’ Animal Care and Use Committees.

**Table 5:** GenBank accession numbers

**Species**	**Gene**	**Accession number**
*O. mykiss*	*mstn1a*	JN990743
	*mstn1b*	JN990751
	*mstn2a*	JN990760
	*mstn2b*	JN990770
*O. tshawytscha*	*mstn1a* (fall)	JN990744
	*mstn1b* (fall)	JN990754
	*mstn1a* (spring)	JN990745
	*mstn1b* (spring)	JN990755
	*mstn2a*	JN990762
	*mstn2b*	JN990772
*O. clarkii*	*mstn1a*	JN990742
	*mstn2a*	JN990758
*O. kisutch*	*mstn1a*	JN990737
	*mstn1b*	JN990738
	*mstn2a*	JN990759
	*mstn2b*	JN990769
*O. keta*	*mstn2b*	JN990768
*O. nerka*	*mstn1b*	JN990752
	*mstn2a*	JN990761
	*mstn2b*	JN990771
*S. salar*	*mstn1a*	EF392862.1/JN990746
	*mstn1b*	AJ316006.2/JN990753
	*mstn2a*	EF392863.1/JN990763
	*mstn2b*	EF392864.1/JN990773
*Sv. Fontinalis*	*mstn1a*	JN990748
	*mstn1b*	AY227655.1/JN990739
	*mstn2a*	JN990764
*C. lavaretus*	*mstn1a*	JN990741
	*mstn1b*	JN990750
	*mstn2a*	JN990757
*C. clupeaformis*	*mstn1a*	JN990740
	*mstn1b*	JN990749
	*mstn2a*	JN990756
*T. arcticus*	*mstn2a*	JN990765
*T. thymallus*	*mstn2a*	JN990766
*P. williamsoni*	*mstn2a*	JN990767
*R. retropinna*	*mstn1*	JN990747
	*mstn2*	JN990774

*C., Coregonus; O., Oncorhynchus; P., Prosopium; R., Retropinna; S., Salmo; Sv., Salvelinus; T., Thymallus;* fall/spring, seasonal runs.

### Gene cloning

A multiple sequence alignment was first constructed using several known fish myostatin cDNA sequences. The consensus sequence was then used to generate PCR primers, some degenerate, suitable for amplifying partial or complete sequences of different myostatin genes. This includes primers specific to individual paralog subfamilies or primers that could presumably recognize conserved sites among all four genes. Many different primer sets were used and a list of primers will be supplied upon request. The specific PCR conditions varied depending upon the primer set used, although in general, 50 ng gDNA was amplified using a high fidelity polymerase (Pfu, Stratagene, http://www.stratagene.com), primer-specific annealing temperatures and a 2 min extension period for each cycle. The PCR products were then sub-cloned into the Topo TA vector (Invitrogen, http://www.invitrogen.com) and sequenced in a university core.

### In silico analysis of gene structure

Complementary DNA (cDNA) sequences for previously cloned MSTN-1a, -1b, -2a, and -2b genes were either downloaded from the National Center for Biotechnology Information (NCBI) or constructed from genomic DNA (gDNA) sequences. When gDNA was used, complete gene structures were identified using GenScan (http://genes.mit.edu/GENSCAN.html) and the exonic sequences were consequently used to construct coding sequences. Initial nucleic acid sequence analyses of cDNA were performed using ClustalW and default parameters in MacVector 10.0.2 (http://www.macvector.com). Coding sequences were similarly analyzed and both required manual editing. The intron splice site consensus sequences of the salmonid MSTN-2 genes were identified by searching for known splice site consensus sequences: 5’ (A or C)AG/GURAGU where R = G or A and 3’ CAG/G. Putative branch points, also necessary for mRNA splicing, were determined by searching for TNCTRAY where N = any nucleotide, R = G or A, and Y = C or T. The number and location of YCAY motifs, regions known to influence spliceosome binding, were also located.

### Phylogenetic analyses

Analyses were performed using cDNA or gDNA collected from 14 different salmonid species and with the common smelt as the outgroup. Sequences were organized into separate files containing MSTN-1 or MSTN-2 genes (a & b paralogs in each) and aligned using MAFFT [43] before testing for optimal substitution using JModelTest [44], all of which fit a General Time Reversible (GTR) model with an estimated gamma distribution. Phylogenetic trees were constructed using a Maximum Likelihood (ML) method and a Bayesian method through the programs PhyML 3.0 [45,46] and MrBayes 3.2 [47], respectively. The accuracy of the resulting topologies was determined through a non-parametric analysis of 1,000 bootstraps or posterior probabilities after 10,000,000 generations [48].

To confirm the systematics of the salmonid lineages, a second phylogenetic analysis was performed using amino acid sequences. Intronic sequences were first located using GENSCAN [49] and GeneMark-E [50] and excised before aligning the resulting sequences with MAFFT and testing for the optimal substitution model with ProtTest3 [51], which resulted in a Jones, Taylor, Thornton (JTT) matrix with an estimated gamma distribution. Phylogenetic trees were then constructed using PhyML 3.0 with a 1,000 bootstrap analysis MrBayes 3.2 with 10,000,000 generations, sampling every 1,000 generations.

Evidence of positive selection was assessed by analysis of dN/dS ratios using Phylogenetic Analysis by Maximum Likelihood (PAML) [52]. Amino acid alignments were coordinated into codons using PAL2NAL [53] in order to estimate ω. Free- and single-ratio estimations of ω were conducted along every branch and the results were compared using a chi-squared analysis to determine evidence for supporting a free-ratios model, which only occurred in the MSTN-2 tree. A phylogenetic tree was then constructed to indicate the dN/dS values for each branch. Values greater than 1 demonstrate branches with high rate variation and thus, possible signals for positive selection. An additional analysis, testing the branch-site model A of PAML, was also performed. Using this model, ω is estimated on a foreground branch against the background branches of the tree to determine rate variation. The results were compared using a *χ*^2^ likelihood ratio test to determine if there was sufficient evidence of supporting the alternative model of an independent ω value for the foreground branch from the rest of the tree over the null model of a fixed ω value of 1 throughout each branch of the tree. A Bayes Empirical Bayes (BEB) analysis was also performed, searching for potential sites within the alignment that were under positive selection. A false discovery rate (FDR) test was performed and the R-based program QVALUE [54] was used to estimate the proportion of true null hypotheses. The program was run using the default settings with a preset FDR of 5%. To further test the occurrence and amount of hybridization and/or incongruent phylogenetic signal that might have occurred between salmonids, a splitstree network was constructed using all four paralogs and the program SplitsTree4 [55].

## Competing interests

DAL is a current section editor for this journal. All of the other authors have nothing to disclose and assert no competing or potential conflicts of interests.

## Authors’ contributions

Experiments were performed by CBL, TN, RAH and VB-V while MFJ and DKG trained CBL and TN and assisted in cloning. The manuscript was written primarily by CBL and TN with editorial assistance from DAL and BDR The latter also planned and supervised these studies and interpreted the data. All authors read and approved the final manuscript.

## Supplementary Material

Additional file 1 **MSTN-1a, -1b amino acid phylogeny.** This phylogeny was created using an amino acid alignment of MSTN-1a and 1b. Introns were excised from the amino acid sequences by the programs GENSCAN and GeneMark-E. The alignments were then made using MAFFT, and the phylogeny was constructed through PhyML 3.0 [45,46], with a 1000 bootstrap analysis. Click here for file

Additional file 2 **MSTN-2a, -2b amino acid phylogeny.** This phylogeny was created using an amino acid alignment of MSTN-2a and 2b. Introns were excised from the amino acid sequences by the programs GENSCAN and GeneMark-E. The alignments were then made using MAFFT, and the phylogeny was constructed through PhyML 3.0 [45,46], with a 1000 bootstrap anlaysis. Click here for file

Additional file 3 **(A) Bayesian Phylogeny of Myostatin proteins (MSTN-1a/1b and MSTN-2a/2b).** The topology was generated using all available myostatin proteins from all for myostatin groups MSTN-1a/1b, and MSTN-2a/2b. The phylogeny was constructed using MrBayes 3.2 [47] with posterior probabilities indicated on the internal nodes of the tree. The tree was rooted with *R. retropinna* as the outgroup. **(B)** Maximum Likelihood Phylogeny of Myostatin proteins (MSTN-1a/1b and MSTN-2a/2b). The topology was generated using all available myostatin proteins from all for myostatin groups MSTN-1a/1b, and MSTN-2a/2b. The phylogeny was constructed using Phyml 3.0 [45,46] with 1,000 bootstraps indicated on the internal nodes of the tree. The tree was rooted with *R. retropinna* as the outgroup. **(C)** Bayesian Phylogeny of Myostatin genes (*mstn1a/1b* and *mstn2a/2b*). The topology was generated using all available myostatin genes from all for myostatin groups *mstn1a/1b*, and *mstn2a/2b*. The phylogeny was constructed using MrBayes 3.2 [47] with posterior probabilities indicated on the internal nodes of the tree. The tree was rooted with *R. retropinna* as the outgroup. Click here for file

Additional file 4 **BEB analysis of MSTN-1a/1b and MSTN-2a/2b.** The amino acid letter represents the amino acid for that position in the first sequence of the alignment used to for the analysis (O. kisutch for MSTN-1a/1b and C. clupeaformis for MSTN-2a/2b). An * indicates there was a gap in the alignment at that position for the first sequence in the alignment. Only sites with probabilities greater than 95% are listed. Click here for file
