# Supplementary figures and images for: The salmonid myostatin gene family: a novel model for investigating mechanisms that influence duplicate gene fate
Source: BMC Evol Biol. 2012 Oct 8;12:202. doi: 10.1186/1471-2148-12-202 (PMC3557186; doi:10.1186/1471-2148-12-202)

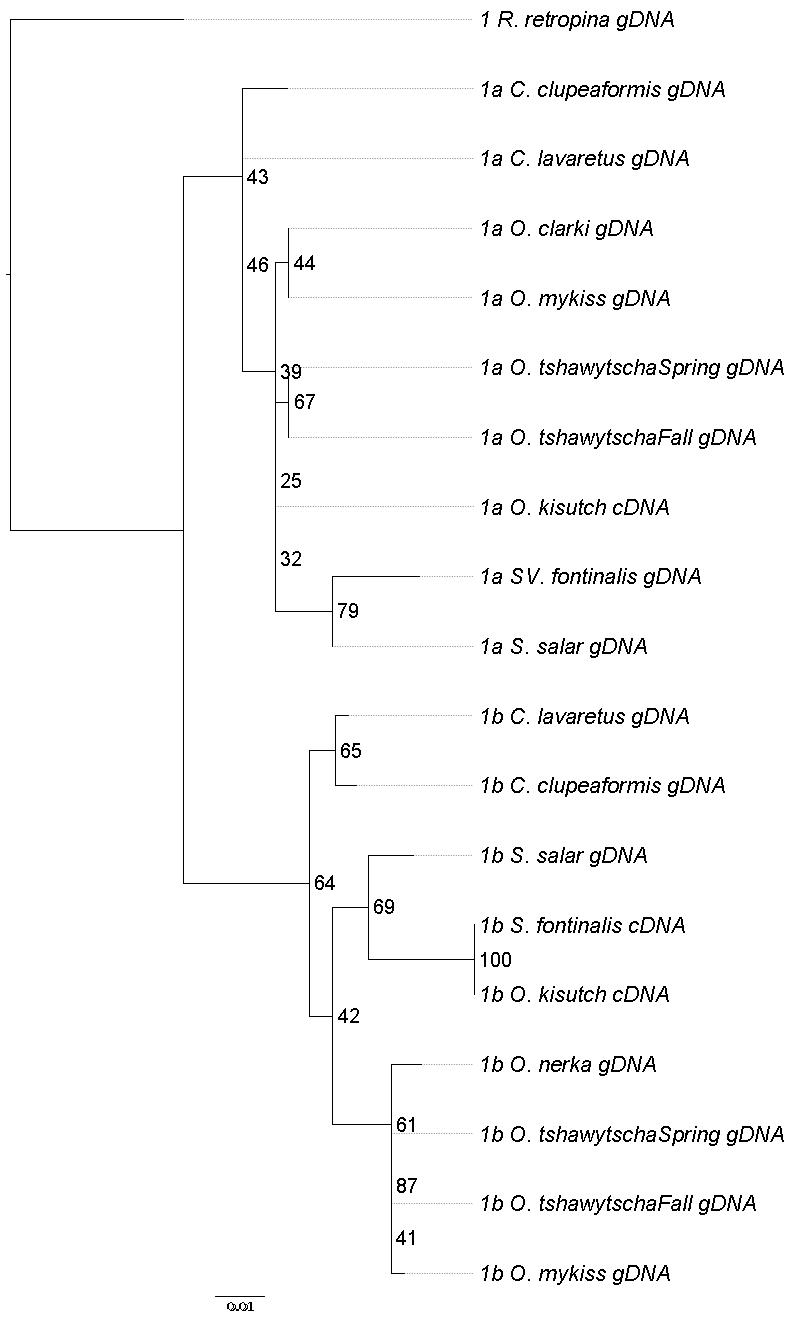

Supplement: Additional file 1 — MSTN-1a, -1b amino acid phylogeny. This phylogeny was created using an amino acid alignment of MSTN-1a and 1b. Introns were excised from the amino acid sequences by the programs GENSCAN and GeneMark-E. The alignments were then made using MAFFT, and the phylogeny was constructed through PhyML 3.0 [45,46], with a 1000 bootstrap analysis. [file 1471-2148-12-202-S1.gif]

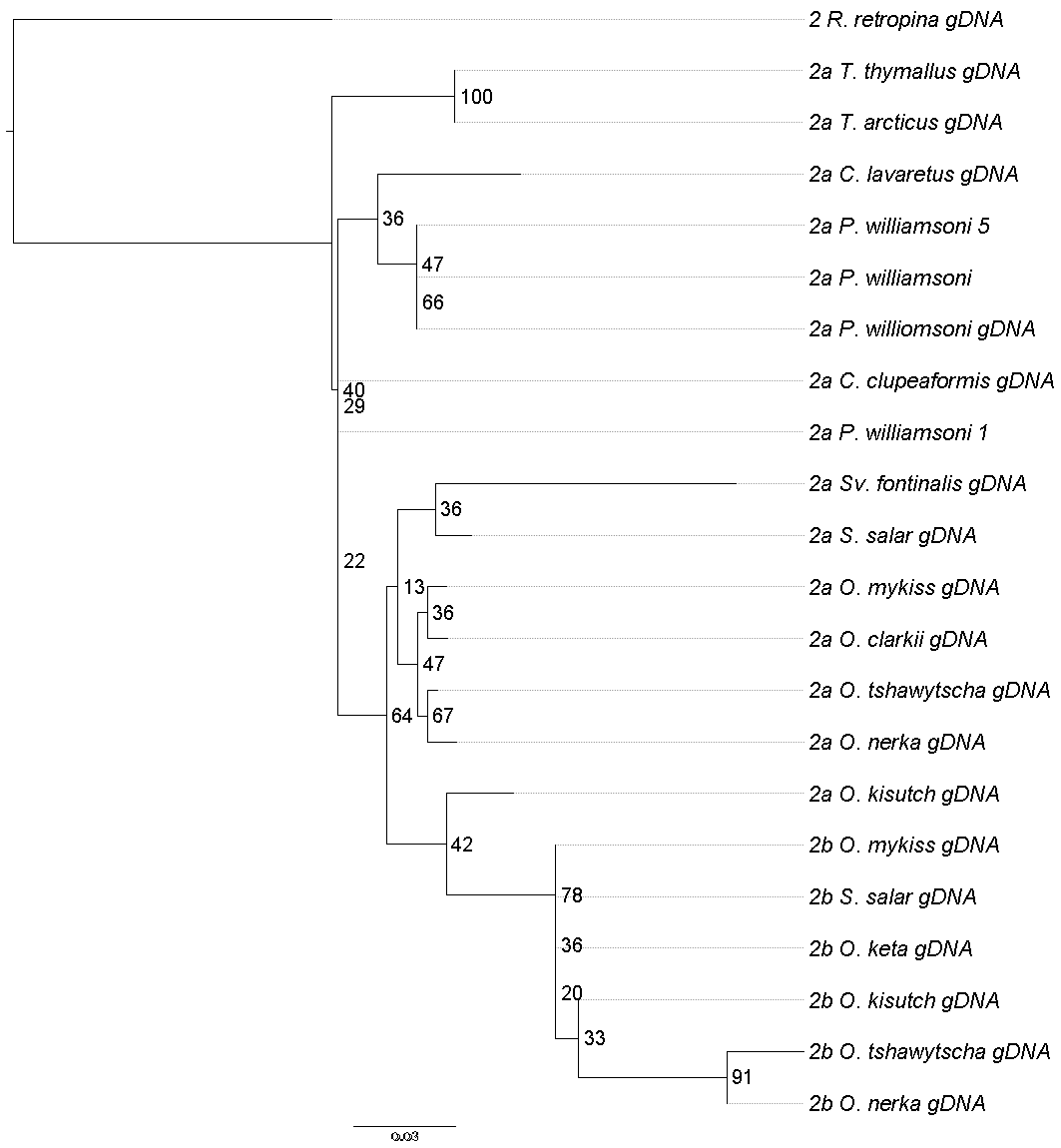

Supplement: Additional file 2 — MSTN-2a, -2b amino acid phylogeny. This phylogeny was created using an amino acid alignment of MSTN-2a and 2b. Introns were excised from the amino acid sequences by the programs GENSCAN and GeneMark-E. The alignments were then made using MAFFT, and the phylogeny was constructed through PhyML 3.0 [45,46], with a 1000 bootstrap anlaysis. [file 1471-2148-12-202-S2.gif]

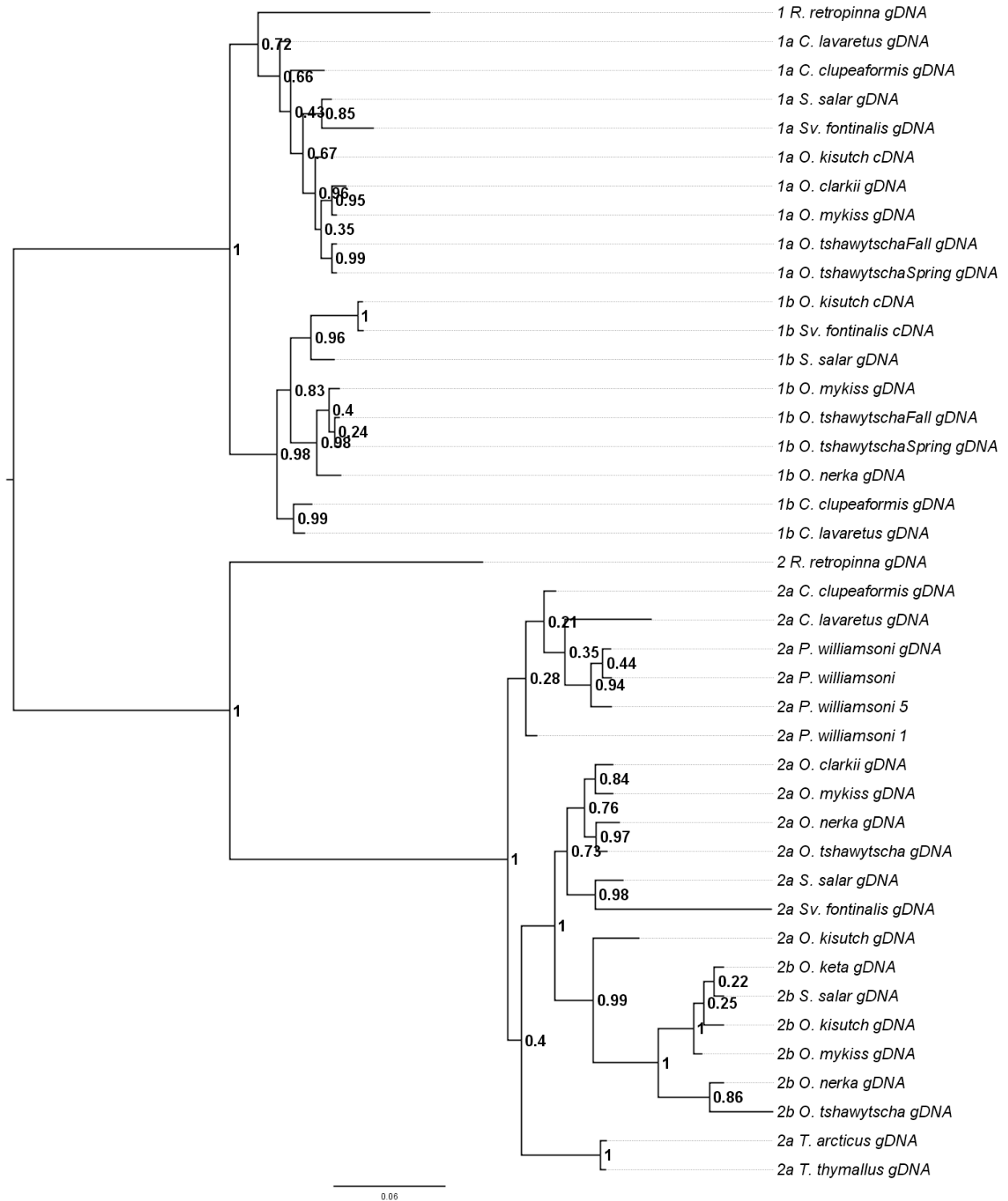

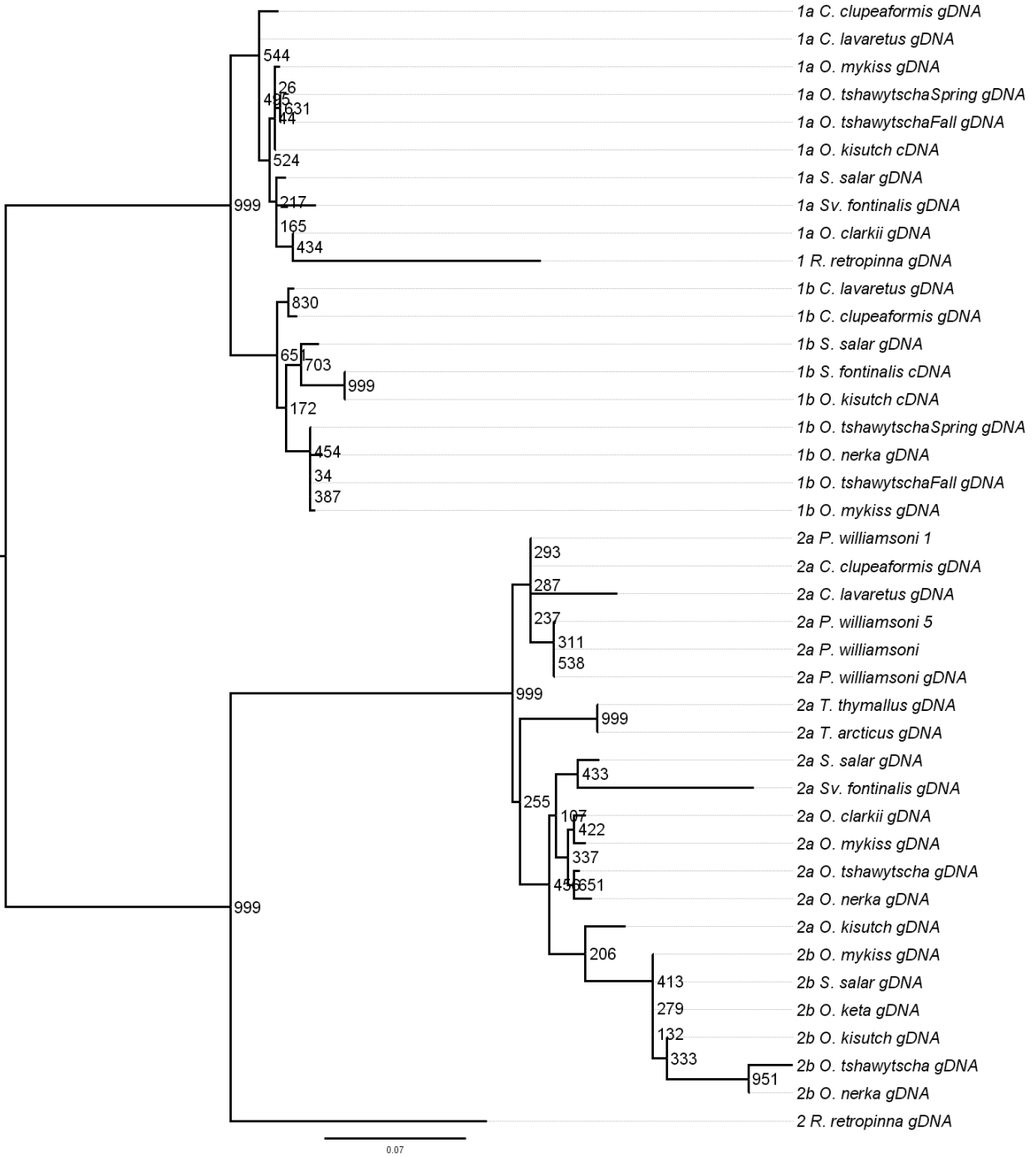

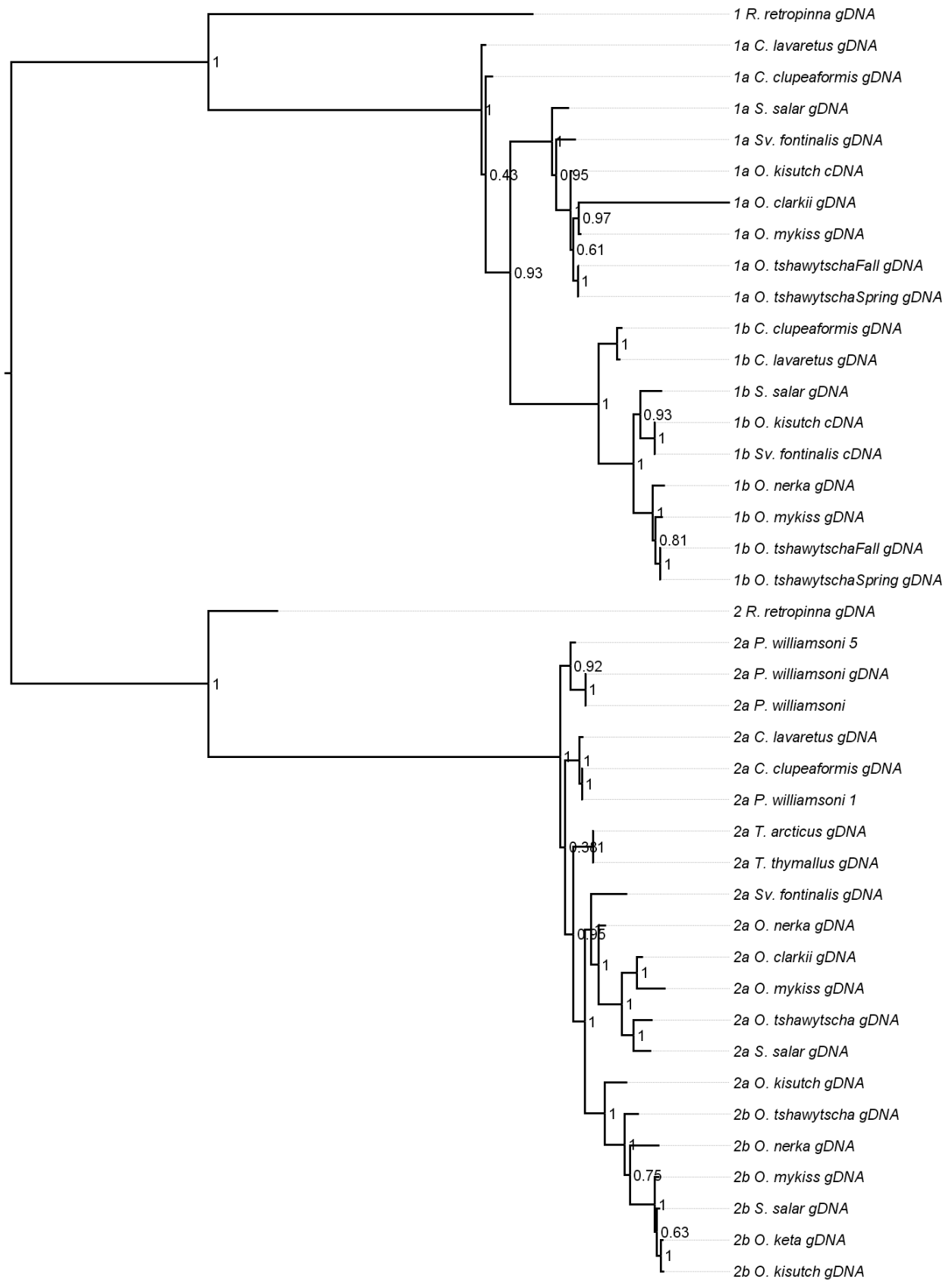

Supplement: Additional file 3 — (A) Bayesian Phylogeny of Myostatin proteins (MSTN-1a/1b and MSTN-2a/2b). The topology was generated using all available myostatin proteins from all for myostatin groups MSTN-1a/1b, and MSTN-2a/2b. The phylogeny was constructed using MrBayes 3.2 [47] with posterior probabilities indicated on the internal nodes of the tree. The tree was rooted with R. retropinna as the outgroup. (B) Maximum Likelihood Phylogeny of Myostatin proteins (MSTN-1a/1b and MSTN-2a/2b). The topology was generated using all available myostatin proteins from all for myostatin groups MSTN-1a/1b, and MSTN-2a/2b. The phylogeny was constructed using Phyml 3.0 [45,46] with 1,000 bootstraps indicated on the internal nodes of the tree. The tree was rooted with R. retropinna as the outgroup. (C) Bayesian Phylogeny of Myostatin genes (mstn1a/1b and mstn2a/2b). The topology was generated using all available myostatin genes from all for myostatin groups mstn1a/1b, and mstn2a/2b. The phylogeny was constructed using MrBayes 3.2 [47] with posterior probabilities indicated on the internal nodes of the tree. The tree was rooted with R. retropinna as the outgroup. [file 1471-2148-12-202-S3.pdf]
