# Supplementary material for: The salmonid myostatin gene family: a novel model for investigating mechanisms that influence duplicate gene fate
Source: BMC Evol Biol. 2012 Oct 8;12:202. doi: 10.1186/1471-2148-12-202 (PMC3557186; doi:10.1186/1471-2148-12-202)
Supplement: Additional file 4 — BEB analysis of MSTN-1a/1b and MSTN-2a/2b. The amino acid letter represents the amino acid for that position in the first sequence of the alignment used to for the analysis (O. kisutch for MSTN-1a/1b and C. clupeaformis for MSTN-2a/2b). An * indicates there was a gap in the alignment at that position for the first sequence in the alignment. Only sites with probabilities greater than 95% are listed. [file 1471-2148-12-202-S4.doc]

**Supplementary Table 1 - BEB analysis of MSTN-1a/1b and MSTN-2a/2b.**

| **MSTN-1a/1b** | | |  | **MSTN-2a/2b** | | |
| --- | --- | --- | --- | --- | --- | --- |
| *Branch* |  | *Positive Sites* |  | *Branch* |  | *Positive Sites* |
| 34 |  | 185 H 0.956 |  | 7 |  | 124 * 0.985 |
| 35 |  | No sites >95% |  |  |  | 125 * 0.995 |
|  |  |  |  |  |  | 147 * 0.999 |
|  |  |  |  |  |  | 148 * 0.987 |
|  |  |  |  |  |  | 192 Q 0.997 |
|  |  |  |  | 18 |  | 41 * 0.996 |
|  |  |  |  |  |  | 42 * 0.994 |
|  |  |  |  |  |  | 50 * 0.996 |
|  |  |  |  |  |  | 51 * 0.979 |
|  |  |  |  |  |  | 52 * 0.997 |
|  |  |  |  |  |  | 53 * 0.996 |
|  |  |  |  |  |  | 55 * 0.997 |
|  |  |  |  |  |  | 56 * 0.988 |
|  |  |  |  |  |  | 57 * 0.996 |
|  |  |  |  |  |  | 85 P 0.955 |
|  |  |  |  |  |  | 89 L 0.955 |
|  |  |  |  | 19 |  | 241 M 0.960 |
|  |  |  |  | 28 |  | No sites >95% |

Letters represent amino acids for the numbered position in the first sequence of the alignment used to for the analysis (*O. kisutch* for MSTN-1a/1b and *C. clupeaformis* for MSTN-2a/2b). An * indicates there was a gap in the alignment at that position for the first sequence in the alignment. Only sites with probabilities greater than 95% are listed.
